# External ventriculostomy-associated infection reduction after updating a care bundle

**DOI:** 10.1186/s12941-023-00612-z

**Published:** 2023-07-15

**Authors:** Mariel Rojas-Lora, Luisa Corral, Ivan Zabaleta-Carvajal, Pau López-Ojeda, Verónica Fuentes-Mila, Iluminada Romera-Peregrina, Cristina Lerma-Briansò, Erika Plata-Menchaca, Alba Pavón, Joan Sabater, Carmen Cabellos

**Affiliations:** 1 Intensive Care Department, Servei de Medicina Intensiva, IDIBELL-Hospital Universitari de Bellvitge, Feixa Llarga S/N, L’Hospitalet de Llobregat, 08907 Barcelona, Spain; 2 Neurosurgery Department, IDIBELL-Hospital Universitari de Bellvitge, Feixa Llarga S/N, L’Hospitalet de Llobregat, 08907 Barcelona, Spain; 3 Infectious Diseases Department, IDIBELL-Hospital Universitari de Bellvitge, Barcelona, Spain; 4 Departament de Ciències Clíniques, Universitat de Barcelona, Av. Mare de Déu de Bellvitge, 3, Feixa Llarga S/N, L’Hospitalet de Llobregat, 08907 Barcelona, Spain; 5 Centro de Investigación Biomédica de Enfermedades Infecciosas (CIBERINFEC, ISCIII), Madrid, Spain; 6 Vall d’Hebron Research Institute, Passeig de La Vall d’Hebron, 129, 08035 Barcelona, Spain; 7 Universitat de Barcelona, L’Hospitalet de Llobregat, Spain

**Keywords:** External ventriculostomy infection, Gram negative bacteria, Care bundle

## Abstract

**Background:**

Despite the clinical benefits of external ventricular drains (EVD), these devices can lead to EVD-related infections (EVDRI). The drainage insertion technique and standardized guidelines can significantly reduce the risk of infection, mainly caused by gram-positive bacteria. However, gram-negative microorganisms are the most frequent causative microorganisms of EVDRI in our hospital. We aimed to determine whether a new bundle of measures for the insertion and maintenance of a drain could reduce the incidence of EVDRI. This cohort study of consecutive patients requiring EVD from 01/01/2015 to 12/31/2018 compared the patients’ characteristics before and after introducing an updated protocol (UP) for EVD insertion and maintenance in 2017.

**Results:**

From 204 consecutive patients, 198 requiring EVD insertion were included (54% females, mean age 55 ± 15 years). The before-UP protocol included 87 patients, and the after-UP protocol included 111 patients. Subarachnoid (42%) and intracerebral (24%) hemorrhage were the main diagnoses at admission. The incidence of EVDRI fell from 13.4 to 2.5 episodes per 1000 days of catheter use. Gram-negative bacteria were the most frequent causative microorganisms. Previous craniotomy remained the only independent risk factor for EVDRI. EVDRI patients had increased mechanical ventilation durations, hospital and ICU stays, and percutaneous tracheostomy requirements.

**Conclusions:**

A care bundle focusing on fewer catheter sampling and more accurate antiseptic measures can significantly decrease the incidence of EVDRI. After implementing the management protocol, a decreased incidence of infections caused by gram-negative and gram-positive bacteria and reduced ICU and hospital lengths of stay were observed.

## Background

External ventricular drains (EVD) are often used in modern neurosurgical practice for the management of acute hydrocephalus, monitoring intracranial pressure, or draining intraventricular blood. Despite their clinical benefits, complications such as EVD-related infections (EVDRI) are outstanding [1–8].

The clinical manifestations of EVDRI are often subtle since patients often present neurological symptoms due to the underlying pathology or sedative medications. Given that cerebrospinal fluid (CSF) leukocyte, glucose, and protein values are not specific to infection, trends in values are more important than absolute values [1]. The main risk factors for EVDRI are patient-related (e.g., male, age, concurrent infections, high intracranial pressure), hospital-related (e.g., length of stay, use of steroids, insertion site), and catheter-related (e.g., duration, manipulation, leaks) [9–13]. Intraventricular hemorrhage, intracranial hypertension, craniotomy, the average time of EVD, and concurrent infections, are the most common conditions associated with EVDRI [14].

Improvement of drainage insertion technique and implementation of standardized guidelines can significantly reduce the risk of EVDRI [2, 14]. However, there is no standard consensus for the management of EVD. Some recent protocols consider chlorhexidine patches to minimize bacterial colonization of catheters [15] and the incidence of ventriculitis [16]. Despite their promising clinical applications, chlorhexidine patches offer limited protection against gram-negative microorganisms [17], the most frequent etiology of EVDRI in our working environment. In this observational study, we aimed to determine whether a new bundle of measures focused on the insertion and maintenance of EVD could reduce the incidence of EVDRI.

## Methods

### Study design and participants

A cohort study was conducted in a 700-bed university hospital for adults in Spain, covering 500,000 people and serving as a referral center for advanced procedures for 2.5 million inhabitants (30% of Catalonia’s population) from the southern Barcelona metropolitan area.

Consecutive patients were included if they were admitted to the neurocritical intermediate care area (6 beds) or intensive care unit (ICU) (36 beds) from 01/01/2015 to 12/31/2018 and required an EVD. A multidisciplinary team (e.g., infectious diseases, neurosurgery, intensive medicine, anesthesiology, and microbiology) performed patient decision-making. We excluded patients < 18 years, with previous EVDRI, or refusing the EVD informed consent. The Clinical Research Ethics Committee of Bellvitge University Hospital (HUB) approved the study (reference PR317/18).

### Protocol, definitions, and variables

Analyses of the incidence of EVDRI and associated risk factors were performed after updating the EVD management protocol (updated protocol, UP) in 2017. We compared patients before (pre-UP) and after (post-UP) the protocol update. The care bundle added to the updated protocol in 2017 is detailed in Table 1 (highlighted in bold). It included staff training and introduced a checklist for protocol systematization. Our unit routinely places conventional drainages first, reserving antibiotic- and silver-covered drainages for patients with suspected infection or at significant risk for developing an EVDRI. The implementation of the updated care bundle coincided with a local campaign to raise awareness of the importance of drainage care and its complications. We held workshops to explain the new measures to improve acceptance and protocol adherence, targeting nursing and medical staff. This was accomplished using a checklist to enhance support for protocol recommendations and ensure rigorous application. Increasing awareness of possible complications and introducing the need to record practices may have contributed to decreasing the number of unnecessary manipulations and sampling while improving adherence to aseptic measures.

**Table 1:** Updated EVD Handling and Insertion Protocol, 2017

Checklist (completed by nursing assistant)	*Before EVD insertion* 1. Informed consent signature 2. Date of insertion 3. Location of insertion (operating room, bedside, or Emergency Department) 4. Operator's Record (Senior or junior Surgeon) 5. Number of people in the room 6. Surgeon (hand washing, use of cap, mask, gloves, and sterile gown, plus **change of gloves after placement of sterile field and before catheter insertion**) 7. Assisting staff (Use of masks, hand washing, and gloves) 8. Patient preparation (wide shaving, washing with soap and water, paint with Betadine, field collation as a sterile blanket, and antibiotic prophylaxis administration): a. Cefuroxime, 1.5 g IV just before implantation as a single dose b. If allergies to cephalosporins, use vancomycin 1 g IV 60 min before the procedure
	*EVD insertion (dressing change procedure)* 1. EVD type 2. Drainage tunneling 3–5 cm from the insertion point 3. Fixation of the catheter with silk 2/03 4. Clean with chlorhexidine spray and connection to the collector system 5. Sterile protection of the first key of the collecting system for subsequent extraction of samples: a. Chlorhexidine spray b. Chlorhexidine-soaked gauze wrap c. Wrap with second protective gauze of the sterile area **6. Cover with chlorhexidine dressing (Tegaderm CHG 3M**^**®**^)
Catheter care	**1. Perform head washing every 4 days with chlorhexidine soap** 2. Healing of the insertion point. Changed every 4 days or whenever it is dirty, wet, or unhooked a. Patient mask placement b. The person performing the cure will wear a mask, wash their hands, and wear sterile gloves c. Use sterile drape and gauze, physiological saline, and chlorhexidine antiseptic solution (clean the insertion point with saline and disinfect the skin with 2% alcoholic chlorhexidine solution) d. Replace the transparent sterile dressing soaked in chlorhexidine (Tegaderm CHG 3M) e. If necessary, use a hair shaver for the area surrounding the drain, and use Nobecutan^®^ and/or Cavilon™ to ensure adherence of the dressing 3. Healing of connections. Assess whenever the connection is used: a. Place a mask on the patient if necessary (not in intubated patients) b. The person who performs the cure will wear a mask, wash their hands, and wear sterile gloves c. Use sterile cloth and gauze with 70º alcohol solution to disinfect the connections and then protect with sterile gauze 4. Collection system change: replace the drainage bag when 3/4 full 5. **Sampling will be done from the 7th day and then every 4 days if there are no signs of infection**, and at any time in case of suspected infection: a. Clamp the catheter 15 min before extraction b. Disinfect the connections with 70º alcohol c. Extract the sample (≤ 5 cc) through the connection most proximal to the catheter, using gentle aspiration d. Use new caps for the 3-way faucets when they are opened

Changes made in the protocol update has been highlighted in bold

Abbreviations: EVD = External ventricular drain; IV = intravenous

EVDRI was defined as the presence of positive CSF cultures, microorganisms on Gram stain, and suggestive symptoms. Cultures were considered for EVDRI if positivity was detected from 24 h after implantation to 5 days after removal [6]. In cases of suspected or unconfirmed EVDRI, patients were evaluated by the multidisciplinary team and the treating team. The criteria used to rule out EVDRI were: (1) absence of symptoms; (2) delayed growth in the CSF culture (e.g., *Corynebacterium* or gram-negative bacilli based on a negative Gram stain); and (3) subsequent negative cultures within 24 h after the initial culture in the absence of empirical or targeted treatment. Given the diagnostic challenge in these cases, patients were followed up until discharge. If EVDRI patient presented another superinfection EVDRI, only the first was analyzed.

We included the following variables: demographic (age, gender, and comorbidities), admission diagnosis, EVD indication, EVD risk factors, EVDRI, ICU and hospital length of stay, mortality, and morbidity. The Glasgow Coma Scale (GCS) was used to assess the neurological status, and the APACHE III (Acute Physiology and Chronic Health Evaluation III) and SOFA (Sequential Organ Failure Assessment Score) scores were used to assess illness severity. Complications of EVD insertion included hematoma, catheter occlusion, or catheter misplacement.

### Statistical analysis

To determine the sample size, we estimated an EVDRI rate of 15 infections per 1000 days of catheter use, expecting the new protocol to reduce this by at least 50%. We accepted an α value of 0.05 to prove a significant reduction in EVDRI. By including 100 patients before (pre-UP) and 100 after (post-UP) the new protocol, the statistical power to detect a significant reduction in EVDI would exceed 80%, indicating the need for a minimum sample size of 200 patients.

We calculated means and standard deviations (SD) or medians and interquartile ranges for quantitative variables and expressed categorical data as frequencies and percentages, as appropriate. Categorical data and proportions were compared with the chi-square test, and continuous variables were compared with Student *t*-tests, Mann–Whitney *U* tests, or Kruskal–Wallis tests. Multivariate logistic regression was performed with significant variables, reporting odds ratios (ORs) and 95% confidence intervals (CIs). An α value of 0.05 was used to determine statistical significance. We analyzed all data in the present study using IBM SPSS statistics 27 (SPSS Inc^©^, Chicago, USA).

## Results

### Participants and demographic characteristics

The study sample comprised 204 consecutive patients (54% females) with a mean age of 55 (SD 15) years who required EVD insertion. In total, 94% of the patients required ICU admission, and 6% required admission to an intermediate care unit, while 65% had undergone neurointervention (39% craniotomy and 26% aneurysm embolization). We excluded one patient with craniotomy infection and five with ventriculoperitoneal drain infection. The final sample included 198 patients.

The demographic characteristics and diagnoses of the patients in the pre-UP group (n = 87) and post-UP group (n = 111) were broadly similar. However, the pre-UP group had fewer cases of intracranial hemorrhage (at admission), craniotomy, or complications during EVD insertion. The pre-up group had an increased number of intracranial tumors and hospital length of stay. Also, they presented different reasons for EVD removal, and worse GCS and SOFA scores. After logistic regression analyses, differences in the SOFA score, complications during EVD insertion, and placement of tunneled catheters were the only independent variables (Table 2).

**Table 2:** Demographic, clinical, and first EVD catheter characteristics

	N = 198	Pre-UP n = 87	Post-UP n = 111	Bivariate	Regression
Age (mean, SD)		53 ± 14	57 ± 15	NS	NS
Gender	Female	56%	53%	NS	NS
Pathological history	Diabetes	18%	12%	NS	
	Hypertension	37%	40%	NS	
	COPD	50%	50%	NS	
	Renal failure	0%	4%	NS	
	Alcoholism	5%	3%	NS	
	Other				
Diagnosis at hospital admission	SAH	40%	45%	0.004	NS
	ICH	15%	31%		
	Tumor	23%	7%		
	TBI	5%	4%		
	VPSD	2%	0%		
	Other	15%	13%		
GCS median (Q1–Q3)	Hospital admission	14 (9–15)	12 (7–15)	0.033	NS
GCS median (Q1–Q3)	ICU admission	10 (6–14)	8 (5–13)	0.031	NS
Severity scales	APACHE III (mean, SD)	52 ± 28	59 ± 24	NS	
	SOFA (median, Q1–Q3)	5 (3–7)	6 (5–9)	0.014	0.04
Previous neuro-intervention		46%	31%	0.027	
Intraventricular hemorrhage		54%	76%	0.001	NS
Concomitant infection		82%	86%	NS	NS
Nasal colonization		3%	2%	NS	
Rectal colonization		22%	13%	NS	
Barbiturate		2%	3%	NS	
Steroids		59%	52%	NS	
Intrathecal urokinase		9%	3%	0.048	NS
Mechanical ventilation (MV)		85%	82%	NS	
Days of MV	Median, Q1–Q3	3 (1–16)	8 (1–18)	NS	
Tracheostomy		28%	30%	NS	
Length of stay in ICU	Median, Q1–Q3	12 (2–25)	9 (2–22)	NS	
Length of stay in hospital	Median, Q1–Q3	39 (19–63)	30 (17–49)	0.018	
GOS at hospital discharge	Dead	24%	27%	NS	
	Vegetative state	1%	1%		
	Alive and conscious	75%	72%		
First EVD catheter characteristics					
Operator	R1	2%	6%	0.032	NS
	R2	13%	20%		
	R3	17%	20%		
	R4	23%	13%		
	R5	10%	5%		
	Neurosurgeon	33%	27%		
	Other hospital or unknown	1%	9%		
Place of insertion	Critical units	52%	55%	NS	
	Ward	3%	3%		
	Operating room	37%	28%		
	Emergency box	8%	12%		
	Other hospital	0%	2%		
Tunneled		59%	97%	0.000	P < 0.001
ICP monitoring		78%	79%	NS	
Prophylaxis		94%	97%	NS	
Complications during insertion	Misplacement/hematoma	25%	12%	0.021	P < 0.001
	No necessary	44%	52%		
	Ventriculoperitoneal shunt insertion	14%	13%		
Reason for catheter removal	Misplacement, obstruction, dysfunction, surgery, or accidental removal	34%	18%	0,007	NS
	Death or withdrawal of care	8%	17%		
Days of EVD		9 (4–15)	12 (7–15)	NS	NS

*APACHE *Acute Physiology and Chronic Health Evaluation, *COPD* Chronic obstructive pulmonary disease, *EVD* External ventricular drain, *GOS* Glasgow outcome scale, *ICH* Intracerebral hemorrhage, *ICU* Intensive Care Unit; *Q1* quartile 1, *Q3* quartile 3, *SAH* Subarachnoid hemorrhage, *SD* standard deviation, *SOFA* Sequential Organ Failure Assessment Score, *TBI* Traumatic brain injury, *VPSD* Ventriculoperitoneal shunt dysfunction, *NS* Not significant

### Occurrence of EVDRI

During the study period, 261 EVD catheters were placed; 198 were first placements and 63 relocations. The incidence of EVDRI decreased from 13.4 per 1000 days of catheter use in the pre-UP group to 2.5 per 1000 days in the post-UP group (Table 3). Of the 198 patients, 138 had negative results in all follow-up cultures, and 60 had at least one positive culture (CSF, EVD catheter, or underlying skin). Among the 60 patients with positive cultures, 37 were not considered as having EVDRI, given that symptoms, CSF, or cultures were not suggestive (17 only had a positive EVD catheter result, and 18 had a negative CSF culture < 24 h after the first or delayed growth with other negative CSF cultures). Thus, 23 patients were considered to have EVDRI, 18/87 (21%) in the pre-UP group and 5/111 (4%) in the post-UP group (Chi-square, < 0.001). Five of 23 EVDRI patients presented superinfection EVDRI (4 Pre-UP and 1 post-UP). Mortality did not change significantly according to infection status (30% vs. 25%) or before and after protocol implementation (24% vs. 28%).

**Table 3:** EVDRI per 1000 days of EVD catheter use

Year	198 patients	EVDRI	Days 261 catheters	Episodes /1000 days of catheter 23/2932 = 7.8
2015	44	10 (23%)	10/745	13.4
2016	43	8 (19%)	8/711	11.2
2017	56	3 (5%)	3/696	4.3
2018	55	2 (4%)	2/780	2.5

EVD = External ventricular drain; EVDRI = EVD-related infection

Among EVDRI patients, first catheters were most commonly involved (17/198, 9%; 13 pre-UP and 4 post-UP), the second catheter in 5 patients (5/47, 12%; 4 pre-UP and 1 post-UP), and the third catheter in one patient (1/13, 8%). Diagnosis relied on CSF alone in most cases (70%), followed by CSF plus EVD catheter culture (26%), and few relied on the EVD catheter alone (4%). EVDRI patients had CSF values showing decreased glucose (80%), increased protein (30%), and increased cell counts (55%). Fever was present in 70%, and neurological impairment was present in 35%. Gram-negative bacteria caused 61% of EVDRI, gram-positive bacteria in 30%, and *Candida albicans* in 9%. In the pre-UP group, 67% were caused by gram-negative bacteria, 22% by gram-positive bacteria and 11% by *Candida albicans.* In the post-UP, 40% were caused by gram-negative bacteria and 60% by gram-positive bacteria (Table 4). Isolated micro-organisms were susceptible to the usual antibiotics with the exception of 1 carbapenem-resistant *Pseudomonas aeruginosa*, 1 *Pseudomonas aeruginosa resistant to* aztreonam, antipseudomonal cephalosporins and penicillin*,* 1 *Escherichia coli* BLEE, 1 *Acinetobacter baumannii* only susceptible to colistin and amikacin and 1 methicillin-resistant *Staphylococcus aureus.*

**Table 4:** Microbiology of the EVDRI PreUp and PostUp

		Pre-UP N = 18/23	Post-UP N = 5/23
Gram-negative bacteria	*Pseudomonas aeruginosa*	3/18	1/5
	*Klebsiella pneumoniae*	3/18	–
	*Escherichia coli*	1/18	1/5
	*Enterobacter cloacae*	3/18	–
	*Acinetobacter baumannii and pseudomonas aeruginosa*	1/18	–
	*Serratia marcescens*	1/18	–
Gram-positive bacteria	*Staphylococcus aureus*		1/5
	*Staphylococcus epidermidis*	3/18	1/5
	*Staphylococcus saccharolyticus*	1/18	–
	*Another gram positive*	–	1/5
Fungi	*Candida albicans*	2/18	–

*EVDRI* EVD-related infection

### Risk factors for EVDRI

We conducted a bivariate analysis of risk factors. Patient characteristics associated with increased risk of infection were previous craniotomy, insertion site infection, concomitant systemic infection, and rectal colonization (Table 5). In the logistic regression with significant variables, the previous craniotomy remained the only independent risk factor, having an OR of 2.7 (95% CI, 1.1–6.8). The characteristics of the 198 first catheters (17 EVDRI) are shown in Table 6.

**Table 5:** Patient-dependent risk factors for EVDRI

		Case No	No EVDRI	EVDRI	Bivariate	Logistic regression
EVD infection		198	n = 175	n = 23		
Age (mean ± SD)			55 ± 15	53 ± 16	NS	
Gender	Female	108	96/175 (55%)	12/23 (52%)	NS	
Diagnosis at admission	Subarachnoid hemorrhage	85	75/175 (43%)	10/23 (44%)	NS	
	Intracerebral hemorrhage	48	44/175 (25%)	4/23 (8%)		
	Tumor	28	23/175 (13%)	5/23 (22%)		
	Traumatic brain injury	8	7/175 (4%)	1/23 (4%)		
	Other	27	25/175 (14%)	2/23 (9%)		
GCS median (IQR)	At hospital admission		13 (7–15)	13 (8–15)	NS	
GCS median (IQR)	At ICU admission		9 (5–14)	9 (7–15)	NS	
Severity scores at ICU admission	Apache III (mean ± SD)		51 ± 26	55 ± 27	NS	
	SOFA median (IQR)		6 (4–8)	6 (4–8)	NS	
Previous open neurosurgery		74	61/175 (35%)	13/23 (57%)	0.043	2.7 (95% CI, 1.1–6.8)
Intraventricular Hemorrhage		131	116/175 (66%)	15/23 (65%)	NS	
Concomitant systemic infection		167	144/175 (82%)	23/23 (100%)	0.028	NS
Nasal colonization		5	4/175 (2%)	1/23 (4%)	NS	
Rectal colonization		33	25/174 (14%)	8/23 (35%)	0.014	NS
Other treatments:	Barbiturate	11	9/175 (5%)	2/23 (9%)	NS	
	Corticosteroids	109	95/175 (54%)	14/23 (61%)	NS	
	Urokinase	5	4/175 (2%)	1/23 (4%)	NS	
Indication for EVD	Hydrocephalus	110	96/175 (55%)	14/23 (61%)	NS	
	Intraventricular hemorrhage	80	72/175 (41%)	8/23 (35%)		
	Other	8	7/175 (4%)	1/23 (4%)		
Hospital days before EVD			0 (0–2)	1 (0–9)	NS	

Concomitant systemic infection referred to respiratory, urinary, catheter, or other infection. Nasal and rectal colonization referred to positive resistant cultures of nosocomial screenings made routinely

*EVD*  External ventricular drain, *EVDRI* EVD-related infection, *IQR* interquartile range, *SD* standard deviation, *NS* Not significant

**Table 6:** Characteristics of first EVD catheters in patients with and without EVDRI

		Case No.	No EVDRI	EVDRI	Bivariate	Logistic regression
First catheter		198	181	17		
Operator	Residents	128	119/181(66%)	9/17 (53%)	NS	
	Neurosurgeon	57	50/181(28%)	7/17 (41%)		
	Missing	13	12/181 (6%)	1/17 (6%)		
Place of insertion	Critical care units	106	97/180 (54%)	9/17 (53%)	NS	
	Ward	6	5/180 (3%)	1/17 (6%)		
	Operating room/theater	61	57/180 (32%)	4/17 (23%)		
	Emergency box	21	18/180 (10%)	3/17 (18%)		
	Missing	3	3/180 (1%)	0/17 (0%)		
EVD catheter type	Conventional	189	173/179 (97%)	16/17 (94%)	NS	
	Antimicrobial-impregnated catheter	3	3/179 (1.5%)	0/17 (0%)		
	Silver catheter	4	3/179 (1.5%)	1/17 (6%)		
Tunneled		160	148/180 (82%)	12/17 (71%)	NS	NS
ICP monitoring		157	141/181 (78%)	16/17 (94%)	NS	
Prophylaxis		189	172/181 (95%)	17/17 (100%)	NS	
Complications during insertion	Misplacement or hematoma	37	31/180 (17%)	6/17 (35%)	0.009	NS
Days of EVD			10 (5–15)	14 (8–21)	NS	
Reason for catheter removal	No necessary	89	87/181(4%)	2/17 (12%)	0.005	NS
	Ventriculoperitoneal shunt insertion	26	24/181 (13%)	2/17 (12%)		
	Misplacement, obstruction, dysfunction, surgery, or accidental removal	57	46/181 (25%)	11/17 (65%)		
	Death or withdrawal of care	26	24/181 (13%)	2/17 (12%)		

*EVD* External ventricular drain, *EVDRI* EVD-related infection, *ICP* intracranial pressure, *NS* Not significant

EVDRI patients had adverse outcomes, such as increased EVD and mechanical ventilation duration, tracheostomy requirement, and prolonged ICU and hospital stay (Table 7). Yet, mortality did not increase significantly.

**Table 7:** ICU admission characteristics in patients with or without EVD infection

		Case No	Non-infected	EVDRI	Bivariate
Total days of EVD	Median (IQR)		12 (7–16)	37 (28–45)	< 0.001
Mechanical ventilation		165	142/175 (81%)	23/23 (100%)	0.023
Days of mechanical ventilation	Median (IQR)		5 (1–16)	13 (3–23)	0.003
Tracheostomy		57	44/175 (25%)	13/23 (57%)	0.002
ICU length of stay	Median (IQR)		9 (2–21)	27 (13–47)	< 0.001
Hospital length of stay	Median (IQR)		29 (17–49)	64 (43–104)	< 0.001
GOS at hospital discharge	Death	51	25.4%	30%	NS
	Vegetative	2	1.2%	0%	
	Conscious	143	73.4%	70%	
Death	Neurological	35 (69%)	30 (74%)	3 (38%)	0.02
	Non-neurological	15 (29%)	13 (26%)	4 (50%)	
	EVDRI	1 (2%)	0%	1 (12%)	

*EVD* External ventricular drain, *EVDRI* EVD-related infection, *GOS* Glasgow Outcome Score, *ICU* intensive care unit, *IQR* interquartile range, *NS* Not significant

## Discussion

The updated EVD management protocol was associated with a decrease in the number of EVDRI. Gram-negative bacteria were the most frequent causative microorganisms. Demographic and diagnostic features of patients were similar before and after the protocol update. Moreover, the only independent risk factor for EVDRI was a previous craniotomy. Patients with EVDRI required more days of mechanical ventilation, tracheostomy requirement, and increased ICU and hospital length of stay.

EVDRI is widely defined as a positive CSF culture with or without evidence of microorganisms on Gram stain and associated with high fever and signs of meningitis [6]. Most authors recommend dynamic CSF analysis [5, 18, 19]. The complexity of these patients also hampers diagnosis because their symptoms could be explained by deterioration secondary to the underlying disease [20–22]. The Center for Disease Control and Prevention does not specify definitions for contamination and colonization, as with other devices. Indeed, CSF inflammation can be secondary to non-infectious causes, such as intraventricular hemorrhage or the neurosurgery itself [2, 6, 8, 20–23]. The lack of consensus makes diagnosis difficult, with an incidence of 10%, ranging from 2 to 27% [6, 12, 24–27]^.^ EVDRI may increase mortality to 15%–20% and lengths of ICU and hospital stay, contributing to increased healthcare costs [9, 28].

Several authors have implemented protocols that achieved decreases in the incidence of EVDRI by 0.4% to 18% [29, 30]. This study found a significant decline in the EVDRI incidence from 23 to 4%, from 2015 to 2018, after implementing an updated EVD management protocol. The 2017 guidelines of the Neurocritical Care Society and Infectious Diseases Society of America recommend the systematic use of care bundles. However, as with other studies, there is no consensus on which bundle elements are essential [2, 25, 31–33]. The main changes implemented in our updated protocol concerned hygiene during EVD insertion, routine maintenance, and proper technique for CSF sampling (e.g., changing gloves after field preparation for catheter insertion, the use of dressings with chlorhexidine patches, a reduced number of sample collections, and head washing every 4 days). Also, fewer EVD manipulations and the implementation of staff re-education were implemented, using a checklist to ensure protocol systematization.

Different interventions have been studied to reduce the incidence of EVDRI. The EVD insertion site contamination can be prevented using an aseptic technique and antibiotic prophylaxis [1, 34, 35]. According to Tunkel et al., no prophylactic EVD changes were made [1] in the absence of infection in this study. The updated care bundle considered the need for continuous reassessment of prolonged antibiotic treatment to prevent multi-resistance [36, 37].

Routine daily sample extraction is not widely accepted, although recommended by some authors [5]. Most studies recommend CSF sampling when infection is suspected [2, 19, 30, 35, 38]. Considering our microbiology patterns, gram-negative bacteria accounted for the most frequent cause of EVDRI. Thus, most infections probably emerged from manipulation. In the updated protocol, routine CSF sampling is performed at the time of placement, on day 7, and every 4 days thereafter, unless EVDRI was suspected or confirmed. This differs from the pre-UP protocol, which considered routine CSF sampling every 48 h and upon suspicion of infection. Decreasing unnecessary sampling leading to excessive manipulation of the EVD, along with the implementation of strict hygiene measures, should be considered essential.

When a dressing becomes loose or soiled, the UP (Table 1) recommends dressing exchange using sterile barriers, cleaning the surrounding area with an antiseptic solution, disinfecting catheter connections, wrapping with sterile gauze, and placing a chlorhexidine patch over the catheter exit site, as reported elsewhere [14]. Following the recommendations of Flint et al., chlorhexidine patches (3M™ Tegaderm™ CHG kit, 3M^©^, Minnesota, United States) were introduced in the UP to cover the catheter exit site after tunneling. These patches are safe and reduce the incidence of infections related to central lines and central nervous system catheters [15, 17, 25], offering protection, especially against gram-positive bacteria [39].

The effectiveness and cost-effectiveness of antibiotic-impregnated EVD are controversial, although some studies have shown they are effective at reducing infection [2, 14, 25, 40, 41]. Silver-coated or antibiotic-impregnated catheters are most effective against gram-positive bacteria [2, 25, 39, 42]. Our protocol included tunneling (3–5 cm from the insertion site) and the systematic use of simple silicone drainages, prioritizing drainage care to decrease EVDRI [43, 44]. This result has significant implications for overall costs, given that antibiotic-impregnated catheters are 3–5 times more expensive than silicone catheters [43].

Over recent years, several studies have shown EVD care bundles reduce EVDRI, though most reports have focused on infection caused by gram-positive bacteria [5, 7, 25, 29, 45, 46]. Still, relatively few studies showed a reduction in EVDRI in areas where gram-negative bacteria are responsible for most cases, as in this study [27, 30].

Other studies have typically associated different risk factors with the development of EVDRI, including a previous craniotomy, intracranial pressure > 20 mmHg, coexisting systemic infection, depressed cranial fractures, CSF fistulas, longer EVD use, frequent device manipulation, intraventricular hemorrhage, and some clinical settings [6, 7, 12–14]. However, this study found that a previous craniotomy was the only statistically significant independent risk factor. EVD-related insertion and maintenance complications (e.g., obstruction, misplacement, hematoma, accidental removal, and dysfunction) may reflect patient neurological complexity, thereby increasing the incidence of EVDRI. Still, no other independent associations were observed in this study. Duration of EVD has been related with higher risk of infection, but in this sample it has not being statistically significant. Although mortality did not differ significantly between patients according to infection status, the higher morbidity was associated with an increased need for mechanical ventilation and prolonged ICU and hospital lengths of stay. Consequently, significant increases in hospitalization costs can be inferred.

## Limitations

Although patient characteristics were similar between the groups, the lack of randomization could have hindered the detection of differences. We did not individually evaluate each protocol measure, which precluded attributing the observed success to a specific intervention. Further, we used retrospective data for the pre-UP group, which may have resulted in missing data.

## Conclusion

An updated care bundle for EVD management, centered on less sampling and strict antiseptic measures during catheter insertion, maintenance, and manipulations, was associated with a reduced incidence of EVDRI caused by gram-negative and gram-positive bacteria. The implementation of the bundle was associated with reductions in ICU and hospital length of stay when staff were adequately trained on protocol adherence.

## Data Availability

The datasets used and analysed during the current study are available from the corresponding author on reasonable request.

## Abbreviations

EVD: External ventricular drains
EVDRI: EVD-related infection
GCS: Glasgow Coma Scale
HUB: Hospital Universitari de Bellvitge
ICU: Intensive care unit
SD: Standard deviations
UP: Updated protocol
